# APT070 (mirococept), a membrane‐localizing C3 convertase inhibitor, attenuates early human islet allograft damage *in vitro* and *in vivo* in a humanized mouse model

**DOI:** 10.1111/bph.13388

**Published:** 2016-01-11

**Authors:** Fang Xiao, Liang Ma, Min Zhao, Richard A Smith, Guocai Huang, Peter M Jones, Shanta Persaud, Attilio Pingitore, Anthony Dorling, Robert Lechler, Giovanna Lombardi

**Affiliations:** ^1^ Division of Transplantation Immunology and Mucosal Biology, MRC Centre for TransplantationKing's College London5th Floor Tower Wing, Guy's Hospital, Great Maze PondLondonSE1 9RTUK; ^2^ Department of Diabetes & EndocrinologyKing's College London2nd Floor, The Rayne Institute, 123 Coldharbour LaneLondonSE5 9NUUK; ^3^ Division of Diabetes & Nutritional Sciences, School of MedicineKing's College London, Guy's HospitalGreat Maze PondLondonSE1 9RTUK

## Abstract

**Background and Purpose:**

A major obstacle to islet cell transplantation is the early loss of transplanted islets resulting from the instant blood‐mediated inflammation reaction (IBMIR). The activation of complement pathways plays a central role in IBMIR. The aim of this study was to test the inhibitory effect of “painting” human islets with APT070, a membrane‐localizing C3 convertase inhibitor, on inflammation evoked by exposure to human serum *in vitro* and by transplantation *in vivo* in a humanized diabetic mouse model.

**Experimental Approach:**

*In vitro,* human islets pre‐incubated with APT070 were exposed to allogeneic whole blood. *In vivo*, similarly treated islets were transplanted underneath the kidney capsule of streptozotocin‐induced diabetic NOD‐SCID IL2rγ^−/−^ mice that had been reconstituted with human CD34^+^ stem cells. Complement activation and islet hormone content were assayed using enzyme‐linked immunosorbent assays. Supernatants and sera were assayed for cytokines using cytometric beads array. Morphology of the islets incubated with human serum *in vitro* and in graft‐bearing kidney were evaluated using immunofluorescence staining.

**Key Results:**

Pre‐incubation with APT070 decreased C‐peptide release and iC3b production i*n vitro*, with diminished deposition of C4d and C5b‐9 in islets embedded in blood clots. *In vivo*, the APT070‐treated islets maintained intact structure and showed less infiltration of inflammatory cells than untreated islets. The pretreatments also significantly reduced pro‐inflammatory cytokines in supernatants and sera.

**Conclusions and Implications:**

Pre‐treatment of islets with APT070 could reduce intra‐islet inflammation with accompanying preservation of insulin secretion by beta cells. APT070 could be as a potential therapeutic tool in islet transplantation.

**Abbreviations**
CR1: complement receptor 1
hu‐NSG: humanized NSG
IBMIR: instant blood‐mediated inflammation reaction
iC3b: inactivated C3b
IEQs: islet equivalents
LDH: lactate dehydrogenase
NSG: non‐obese diabetic/severe combined immunodeficiency/interleukin‐2 receptor γ‐chain knockout
sCR1: soluble complement receptor 1 (sCR1)
UCB: umbilical cord blood

## Tables of Links

**Table:** 

**TARGETS**
**GPCRs**
C3a receptor

**LIGANDS**
C3a, complement component C3a
Carbachol

These Tables list key protein targets and ligands in this article which are hyperlinked to corresponding entries in http://www.guidetopharmacology.org, the common portal for data from the IUPHAR/BPS Guide to PHARMACOLOGY (Pawson *et al.*, 2014) and are permanently archived in the Concise Guide to PHARMACOLOGY 2013/14 (Alexander *et al.*, 2013).

## Introduction

Type 1 diabetes (T1D) is characterized by permanent destruction of insulin‐producing beta cells in the pancreatic islets. Transplantation of islets of Langerhans via intra‐portal injection is a promising treatment procedure for patients with severe T1D (Robertson, 2010). However, despite recent progress, the procedure is associated with massive tissue loss caused by an inflammatory reaction, referred to as the instant blood‐mediated inflammatory reaction (IBMIR) (Nilsson *et al.*, 2011). It is believed that complement attack is a major cause of the damage to the islets that occurs during the IBMIR (Tjernberg *et al.*, 2008) and the reaction involves rapid activation of the coagulation and complement system, activation and binding of platelets to the islet surface, and infiltration of leukocytes into the islets (Nilsson *et al.*, 2011). The complement cascade therefore makes a logical therapeutic target for the reduction of graft rejection (Farrar and Sacks, 2014).

The central step in the complement cascade is the cleavage of C3 to C3b by C3 convertase. This leads ultimately to the assembly of the membrane attack complex (MAC) and the release of soluble C3a and C5a, which in turn activate and recruit inflammatory cells (Sacks *et al.*, 2009). Once C3 is deposited covalently as C3b on a membrane, it has two fates. The first is an amplification step, in which more C3 is cleaved and bound to the membrane. This process involves a further complement protein, factor B. When factor B binds to C3b, it is activated by the complement enzyme factor D and forms the C3 convertase enzyme C3bBb. This enzyme cleaves more C3, causing many more C3b molecules to be deposited on the membrane. The second possible fate of bound C3b is catabolism to iC3b (inactivated C3b) and C3dg, creating adhesive contact with corresponding receptors on leucocytes through the complement receptors, CR1‐4, particularly CR3 and CRIg (Walport, 2001a; Walport, 2001b). This step is mediated by regulators that inactivate complement binding to C3b thus promoting its removal and degradation from the enzyme complexes that convert C3 and C5 into their active forms. The regulators exist as membrane bound (e.g. CD35, CD46 and CD55) and soluble (e.g. Factor H) proteins. APT070, also known as Mirococept, is a modified fragment of the complement receptor 1 (CR1, CD35) (Mossakowska *et al.*, 1999; Smith, 2002). It consists of the first 3 consensus domains of the human CR1 and a membrane‐interacting synthetic peptide, which mediates binding to phospholipids on the cell surface and therefore protects the cell against complement activation (Smith and Smith, 2001; Smith, 2002). APT070 showed inhibition of complement and neutrophil activation during cardiopulmonary bypass in man *in vitro* (De Silva *et al.*, 2006), and of the inflammatory responses that follow intestinal (Souza *et al.*, 2005) and renal (Patel *et al.*, 2006) ischaemia and reperfusion injury in rat models. These studies have prompted clinical investigation of the utility of APT070 in man for ischaemia‐reperfusion injury during renal transplantation (Smith, 2007). These properties suggested the possible use of APT070 in protecting human islet transplantation.

The development of mice that are ‘humanised’ by engraftment of human tissues, haematopoietic stem cells or peripheral‐blood mononuclear cells (PBMC), provides an opportunity to study human biological processes *in vivo* that would otherwise not be possible (Shultz *et al.*, 2007). We have recently described human islet allograft rejection in the CD34^+^ stem cell‐reconstituted humanized mouse model (Xiao *et al.*, 2014). Although islets were placed underneath the kidney capsule, rejection was associated with C3b deposition and we suggested that complement may be involved in the recruitment and activation of innate inflammatory cells. We therefore hypothesised that inhibition of complement activation might enhance islet survival. The present study aimed to investigate whether APT070 could protect human islets from complement‐mediated destruction *in vitro*, and attenuate the immune response to human islet allograft *in vivo* in the humanized mouse model (Xiao *et al.*, 2014).

## Methods

### Preparation of human islets

**Figure 1:**
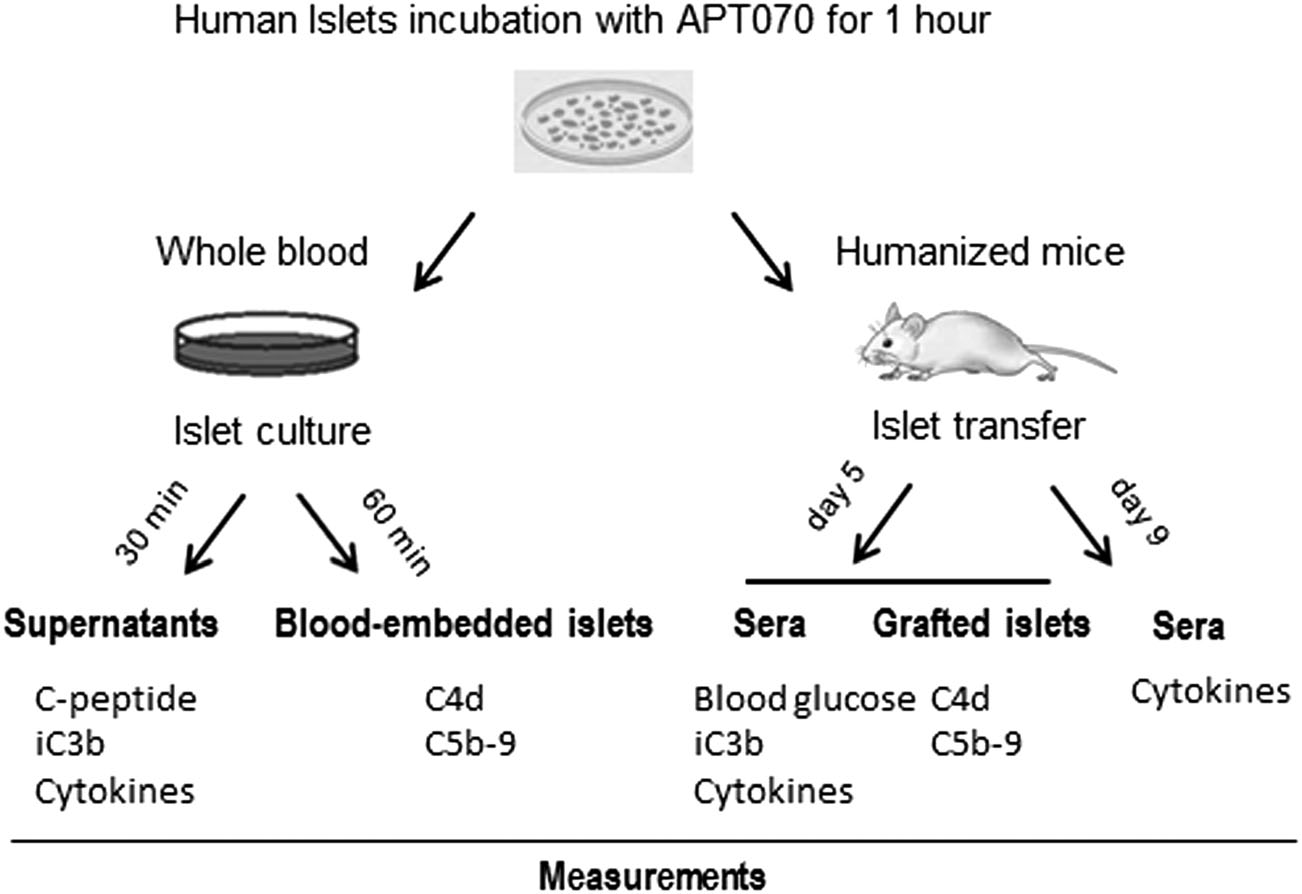
Schematic description of the *in vitro* model mimicking IBMIR, and *in vivo* model of humanized NSG mice.

The procurement and use of all human tissues were with signed informed consent, in accordance with the Declaration of Helsinki and approval from the Institutional Review Board of King's College London. Human islets were obtained from King's Cell Isolation Unit, London, UK. Freshly isolated human islets were incubated with serial dilutions of membrane‐localizing complement regulator APT070 [0.4–1.6 μM; 3000–4000 human islet equivalents (IEQ) mL − 1], or its control molecule APT154 at the equivalent molar concentration, in CMRL 1066 culture medium (Invitrogen) containing 2.5% human albumin at 37 °C in an atmosphere of 95% air 5% CO2 for one hour (Figure 1). The recombinant non‐tailed CR1 fragment APT154 possesses chemical and complement inhibitory properties identical to those of APT070, but is untagged and therefore does not contain the membrane‐binding moiety (Banz *et al.*, 2007). This control molecule was prepared using the cloning and expression strategy as previously described (Dodd *et al.*, 1995; Mossakowska *et al.*, 1999; Smith, 2002). To detect the bound APT070, the treated islets were incubated with a rabbit polyclonal antibody raised against recombinant SCRs (1–3) of CR1 (APT3677, 100 μg mL − 1) (Dodd *et al.*, 1995). Antibody APT3677 was a product of Adprotech Ltd. (Saffron Walden, UK) and is available for research purposes from King's College London (Dr. Richard A Smith). After washing, tetramethylrhodamine isothiocyanante (TRITC)‐conjugated goat anti‐rabbit IgG was applied for one hour at room temperature. Images were acquired using a Cooled Mono14 Bit camera (Q IMAGING, Canada) and Micro‐Manager 1.3 software (University of California, USA).

### In vitro viability and functional assessment of APT070‐treated islets

APT070‐treated islet viability was assessed by a fluorometric viability assay, using fluorescein diacetate and propidium iodide (Sigma, Dorset, UK) (Hansen *et al.*, 1989; London *et al.*, 1989), and kits of the CellTiter‐Glo Luminescent Cell Viability Assay (Promega, Southampton, UK) and lactate dehydrogenase (LDH) assay, according to the manufacturers' instruction. For measuring the dynamics of insulin release, islets were transferred into chambers (50 islets per chamber) of a temperature‐controlled multichannel perifusion system as previously described (Jones *et al.*, 1989). The loaded islets were subsequently perifused (0.5 ml min^−1^) with 2 mM glucose for 10 min and 20 mM glucose for 20 min in Gey's balanced salt solution [0.05% (*w*/*v*) BSA, 2 mM CaCl_2_, 111 mM NaCl, 5 mM KCl, 27 mM NaHCO_3_, 1 mM MgCl_2_, 0.22 mM KH_2_PO_4_ and 0.28 mM MgSO_4_], followed by an additional perifusion of 500 μM carbachol for 18 min. Sampling was performed at 2 min intervals. In another experiment, the extracts of islet pellets were prepared using acid‐ethanol. Insulin content was assessed by radioimmunoassay (RIA) using in‐house assay, as described previously (Jones *et al.*, 1988).

### In vitro model of allogeneic blood‐islet combinations

Approximately 2500 IEQs APT070‐treated islets with a purity of 60–80% were re‐suspended in 1 ml of culture medium and placed in 35‐mm polystyrene petri dishes (BD). One millilitre of freshly drawn human whole blood, ABO blood group typed for compatibility with the islet donor, was added and incubated in 37 °C for 30 min. Supernatants were then collected, with the addition of EDTA (10 mM final concentration) to prevent further complement activation (Mollnes *et al.*, 2002). These were immediately stored with protein inhibitor cocktail (Sigma) at ‐80C for future use.

### Humanized mouse model with APT070‐treated islet transplantation

#### Reconstitution of mice with human stem cells

All animal care and experimental procedures complied with the institutional guidelines and the Home Office Animals Scientific Procedures Act (1986) (UK) and were approved by the Ethical Review Process Committee, King's College London, UK (Project no: 70/7302). These studies comply with the recommendations on experimental design and analysis in pharmacology (Curtis *et al.*, 2015) and the animal studies follow the ARRIVE guidelines (McGrath & Lilley, 2015; McGrath *et al.*, 2015). All animal studies were randomized and observations were made without knowledge of the treatments.

Non‐obese diabetic/severe combined immunodeficiency/interleukin‐2 receptor γ‐chain knockout (NOD/SCID/IL2Rγ^−/−^, referred to here as NSG; The Jackson Laboratory) mice were bred and maintained in the Biological Services Unit of King's College London under specific pathogen‐free conditions. Umbilical cord blood (UCB) was obtained from full‐term normal deliveries. UCB‐derived CD34^+^ stem cells were isolated as described by Xiao *et al.* (2014). Briefly, mononuclear cells were isolated by Ficoll‐Paque (GE Healthcare, Hatfield, UK) gradient separation and enriched for CD34^+^ cells using positive isolation kit according to the manufacturer's instructions (Miltenyi Biotech, Surrey, UK). Mice (6–8 weeks old) were gamma‐irradiated (240 cGy) and then intravenously injected with 2 x 10^5^ CD34^+^ stem cells (referred to as hu‐NSG mice)**.**

#### Flow cytometric analysis of human haematopoietic engraftment

Tail bleeding was performed in hu‐NSG mice 12–16 weeks after injection of CD34^+^ cells. Blood cells were prepared for flow cytometry as previously described (Xiao *et al.*, 2014). Fluorochrome‐coupled antibodies specific for human CD45 were used for detection of human leukocytes. Flow cytometric data were acquired using a FACS Calibur (BD Biosciences, San Jose, CA, USA) and analysed using FlowJo 7.5 software (TreeStar Inc.).

#### Islet transplantation into diabetic ‘humanized' NSG mice

NSG and hu‐NSG mice were rendered diabetic (blood glucose level ≥ 20 mM) by a single i.p. injection of streptozotocin (180 mg kg^−1^) and transplanted with human APT070‐treated islets (IEQs, 3000–4000) under the left kidney capsule as previously published (Xiao *et al.*, 2014). During the surgery, the recipient animals were anaesthetized using 3% isoflurane in a stream of 100% O2 (2 l min^−1^), and were maintained using 1.5–2.0% isoflurane in a stream of 100% O2 (2 l min^−1^). The animals were placed on a heat pad to keep body temperature at 37 °C throughout the procedure. Blood glucose concentration of recipient mice was monitored every 24 hours at the first week and twice a week thereafter using a blood glucose sensor (Abbott Diabetes Care Ltd., Witney, Oxon, UK). Reversal of hyperglycaemia was defined as non‐fasting blood glucose concentration ≤ 13.8 mM (King *et al.*, 2008). NSG mice with successful transplants were subjected to unilateral left nephrectomy to evaluate a return to hyperglycemic state. For islet‐transplanted diabetic hu‐NSG mice, sera were collected by tail sampling at day 5 and day 9 after transplantation, and kept at −80 °C prior to use for further test. The animals were sedated with CO_2_ and killed by decapitation at the end of experiments.

#### Enzyme‐linked immunosorbent assay (ELISA) for human insulin, C‐peptide and iC3b

Supernatant insulin and C‐peptide concentrations were assayed using ELISA kits (Millipore, Watford, UK). ELISA kit for human iC3b (Pathway Diagnostics Ltd., Surrey, UK) was used to determine levels of soluble complement activation product iC3b in supernatant and sera, according to the manufacturer's instructions. All samples were tested in triplicate in the assays.

### Cytokine detection (beads array)

Human cytokines in supernatants and sera were assayed using a human Th1/Th2 11plex kit (eBiosciences) according to the manufacturer's protocol. Data acquisition was performed on a FACS Calibur (BD Biosciences) and analysed using The FlowCytomix Pro 3.0 Software from eBioscience (Xiao *et al.*, 2014).

### Histology analysis

Deposition of activated complements was examined in the islets embedded in blood clots after 60 min incubation with blood, and the harvested graft‐bearing kidney at day 5 after transplantation. The samples were fixed in 10% buffered formalin, and embedded in paraffin. Sections (5 μm) were processed with double immunofluorescence staining. After antigen retrieval by microwaving for 5 min in 0.01 M citrate buffer (pH 6.0), sections were blocked with 10% goat normal serum for 30 min and then incubated overnight at 4 °C with primary antibodies: rabbit anti‐human insulin (1:300; Bioss, polyclonal), mouse anti‐human C4d (1:25; Abcam, clone LP69), mouse anti‐human C5b‐9 (1:100; Abcam, clone aE11), mouse anti‐human CD11b (1:50; eBiosciences, clone ICRF44) and mouse anti‐human CD66b (1:50; BioLegend, clone G10F5). Secondary antibodies FITC‐conjugated goat anti‐mouse IgG and TRITC‐conjugated goat anti‐rabbit IgG (both Sigma) were applied for 2 hours at room temperature (Xiao *et al.*, 2014). Negative controls with non‐specific IgG were processed in parallel. Images were acquired using a Nikon Eclipse Ti‐E Inverted microscopy with 4 x GaAsP Detectors and A1R Si Confocal system with a 60x objective lens and analysed by NIS‐Elements Advanced Research imaging analysis software (Nikon, Surrey, UK). Where the infiltrated cells were quantified, each sample from an individual animal (*n* = 5 animals) was assessed and analysed by using ImageJ software. The deposition of complement was quantified by measuring the mean fluorescence intensity (MFI) in the region of interest area.

### Data analysis

Data are presented as means ± SD. All statistical analysis was performed using the two‐tailed Student's t‐test or two‐way ANOVA analysis, followed by Bonferroni post hoc tests. *P* values <0.05 were considered significant.

### Materials

APT070 and APT 154 were supplied by Adprotech Ltd. (Saffron Walden, UK); carbachol and streptozotocinwere supplied by Sigma (Dorset, UK).

## Results

### Human islets display physiological responses after treatment with APT070

**Figure 2:**
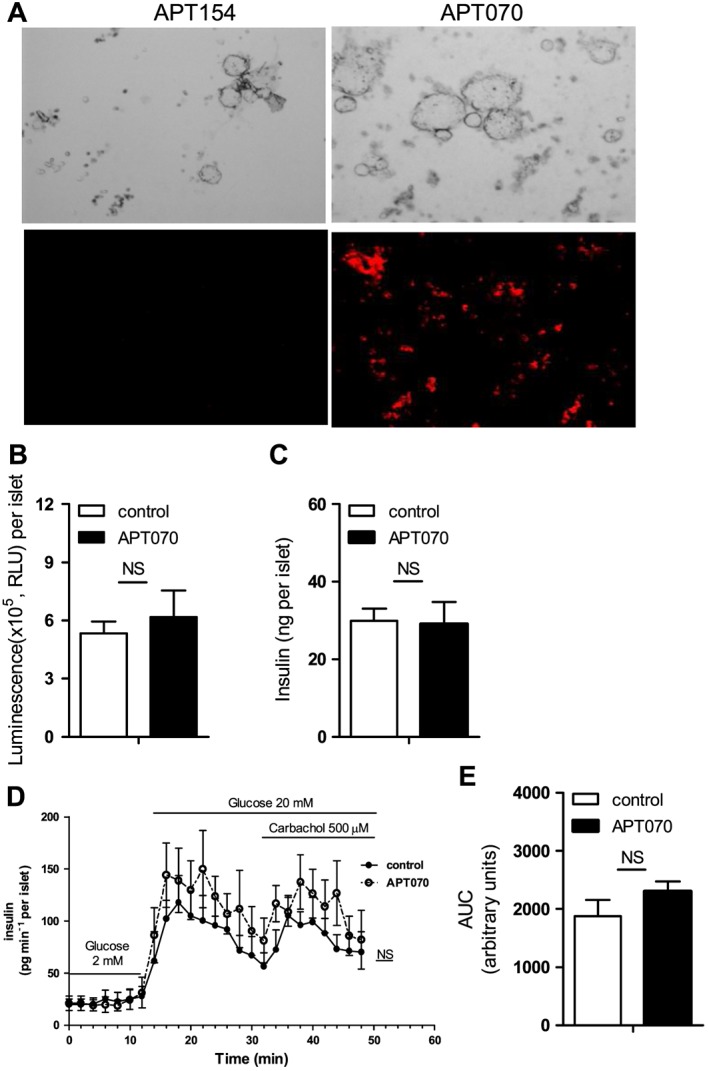
Assessment of islet viability and secretion function**.** (A) Binding of APT070 (0.4 μM) to human islets one hour after incubation. Positive binding is shown in red. Amplifier power: × 60. (B) Islet viability assay. Luminescent outputs, which correlated with islet cell numbers, were measured in APT070 treated and control islets. Values represent the mean ± SD, *n* = 6 (wells of 96‐well plate). (C) Intracellular insulin content in APT070‐treated and control islets. The data are expressed as means ± SD, *n* = 6. (D, E) The dynamics of insulin release. Data are shown as means ± SD, *n* = 8 perifusion channels. RLU: relative luminescence units. AUC: area under curve. Control islets received no treatment. NS: no significant difference.

To ensure that APT070‐treated islets were viable and physically identical to the freshly prepared islet, we examined the effects of APT070 on the viability of human islets. Human islets were incubated in vitro for one hour in culture medium alone (control group) or with APT070 and its control molecule APT154 (Figure 1). The data demonstrate that APT070, but not APT154 bound to the islet (Figure 2A). Dose‐titration of APT070 from 0.4 to 1.6 μM (25–100 μg mL − 1) showed that the lowest dose (0.4 μM) treatment had no effect on islet viability in the fluorescein diacetate and propidium iodide staining (Supplementary Figure S1). This was confirmed by cell viability assay based on quantitation of the APT present (Figure 2B) and the measurement of intracellular content of insulin (Figure 2C). Therefore APT070 was used at a concentration of 0.4 μM (25 μg mL − 1) in further studies. It should be noted that a concentration of 10 μg mL − 1 of APT070 has been used in perfusion experiments in human renal transplantation without apparent adverse effects (Smith *et al.*, 2007).

To evaluate islet function after incubation with APT070, measurements of the rate and pattern of insulin secretion from perifused islets (Figure 2D) confirmed that APT070 pre‐treatment did not inhibit insulin secretory responses to glucose, nor to the receptor‐mediated agonist, carbachol. Expressing the cumulative total insulin secretion during the perifusion as area under the curve (AUC) showed that APT070 pre‐treatment had no significant effects on insulin secretion (Figure 2E), confirming that the β‐cells remained functionally viable after treatment.

### Pre‐incubation of human islet with APT070 prevents the production of activated complement in vitro and in vivo

**Figure 3:**
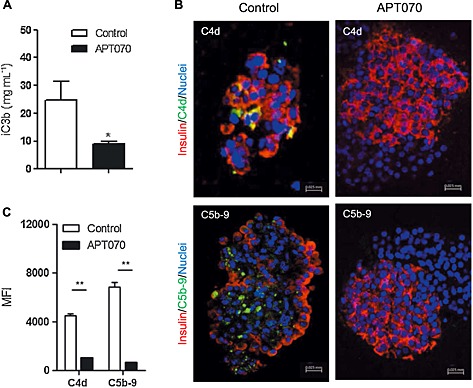
iC3b in supernatants and deposition of C4d and C5b‐9 in blood clot‐embedded islets. (A) Supernatants were collected 30 min after islet exposure to allogeneic whole blood and were assayed for iC3b level by ELISA, *n* = 6. (B) Freshly isolated islets were pre‐incubated with APT070 (0.4 μM) or with culture medium alone (control) for one hour, following by exposed to allogeneic whole blood for one hour. Blood clot‐embedded islets were stained with antibodies specific for insulin and C4d or C5b‐9 molecules. Images are representative from 6 sections in each group. (C) Quantitative analysis of blood‐embedded islet immunostaining. Staining for C4d and C5b‐9 is shown in green. Nuclei were stained with DAPI (blue). Yellow indicates co‐localization of insulin and C4d/C5b‐9. Data are expressed as means ± SD. *: *P* < 0.05; **: *P* < 0.01; significantly different as indicated. MFI: mean fluorescence intensity.

To test whether APT070 has an inhibitory effect on complement activation, islets were exposed to whole blood for 30 minutes. The level of iC3b was significantly decreased in the supernatant of APT070 treated‐islets, compared with control groups (*n* = 6 for each group) (Figure 3A).

To further assess the *in vitro* inhibitory effects of APT070 on complement activation, C4d deposition, as an indicator of complement activation, and C5b‐9, which may cause cell lysis in the blood‐exposed islets, were examined. Pre‐treatment of islets with APT070 almost abolished deposition of both C4d and C5b‐9 in the islet exposed to blood for 60 minutes (Figure 3B, C).

**Figure 4:**
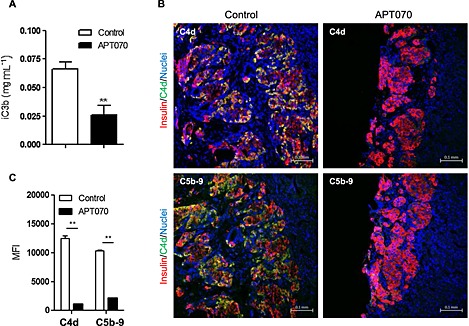
Serum iC3b levels (A) and deposition of C4d and C5b‐9 in islet graft bearing kidney. Sera (A) and islet graft‐bearing kidneys (B) were collected from humanized NSG mice 5 days after islet transplantation. (C) Quantification of the deposition of C4d and C5b‐9 in the graft‐bearing kidney. Islets were pre‐incubated with APT070 for one hour before surgery. Staining for C4d and C5b‐9 are shown in green. Nuclei were stained with DAPI (blue). *n* = 5 mice for each group. Data are expressed as means ± SD. Control islets were incubated with culture medium alone. **: *P* < 0.01; significantly different as indicated. MFI: mean fluorescence intensity.

To evaluate the *in vivo* effects of APT070 on human allograft, humanized mice were generated (hu‐NSG mice) as previously described (Xiao *et al.*, 2014). Immunodeficient mice (NSG) of 4–6 weeks old were engrafted with UCB‐derived CD34^+^ stem cells and, after 16 weeks, a significant percentage of human CD45^+^ cells was detectable in the blood of these mice (Supplementary Figure 2). APT070‐treated islets were transplanted under the kidney capsule of hu‐NSG mice. Although the kidney capsule is not a site where classical IBMIR occurs, deposition of human C3 on human islets implanted beneath the kidney capsule of hu‐NSG mice, associated with circulating human C3, has previously been shown (Xiao *et al.*, 2014), therefore it is appropriate to assess the effect of APT070 on these parameters. The level of human iC3b was found to be decreased in the sera of mice receiving APT070 treated‐islet at day 5 after transplantation, compared with controls (Figure 4A). Furthermore, there was very little deposition of C4d and C5b‐9 in the islet allografts in the APT070 treated group. In contrast, allografts from hu‐NSG mice that had received untreated‐islets exhibited an abundant staining for C4d and C5b‐9 (Figure 4B). There was a significant difference between the two groups (Figure 4C).

### APT070 reduces human islet destruction in human blood in vitro and in islet allografts in humanized NSG mice

**Figure 5:**
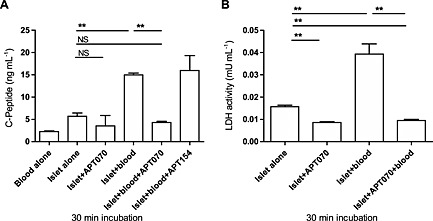
C‐peptide and LDH levels in supernatant from co‐culture of human islet and allogeneic whole blood. The supernatants were sampled 30 min after exposure of human islets to allogeneic whole blood. Data are means ± SD of 6 Eppendorf tubes from two independent experiments. **: *P* < 0.01; significantly different as indicated. NS: no significant difference. Control islets had no treatment.

To evaluate the damage in islets exposed to blood *in vitro*, the release of C‐peptide and of LDH were assessed as indicators of beta cell damage. Measurements of C‐peptide and LDH in supernatants 30 min after incubation of untreated islets with human blood showed significant increases, consistent with islet cell damage (Figure 5). However, the release of C‐peptide and LDH was completely inhibited in the APT070‐treated group, demonstrating protection from blood‐mediated islet cell damage (Figure 5).

**Figure 6:**
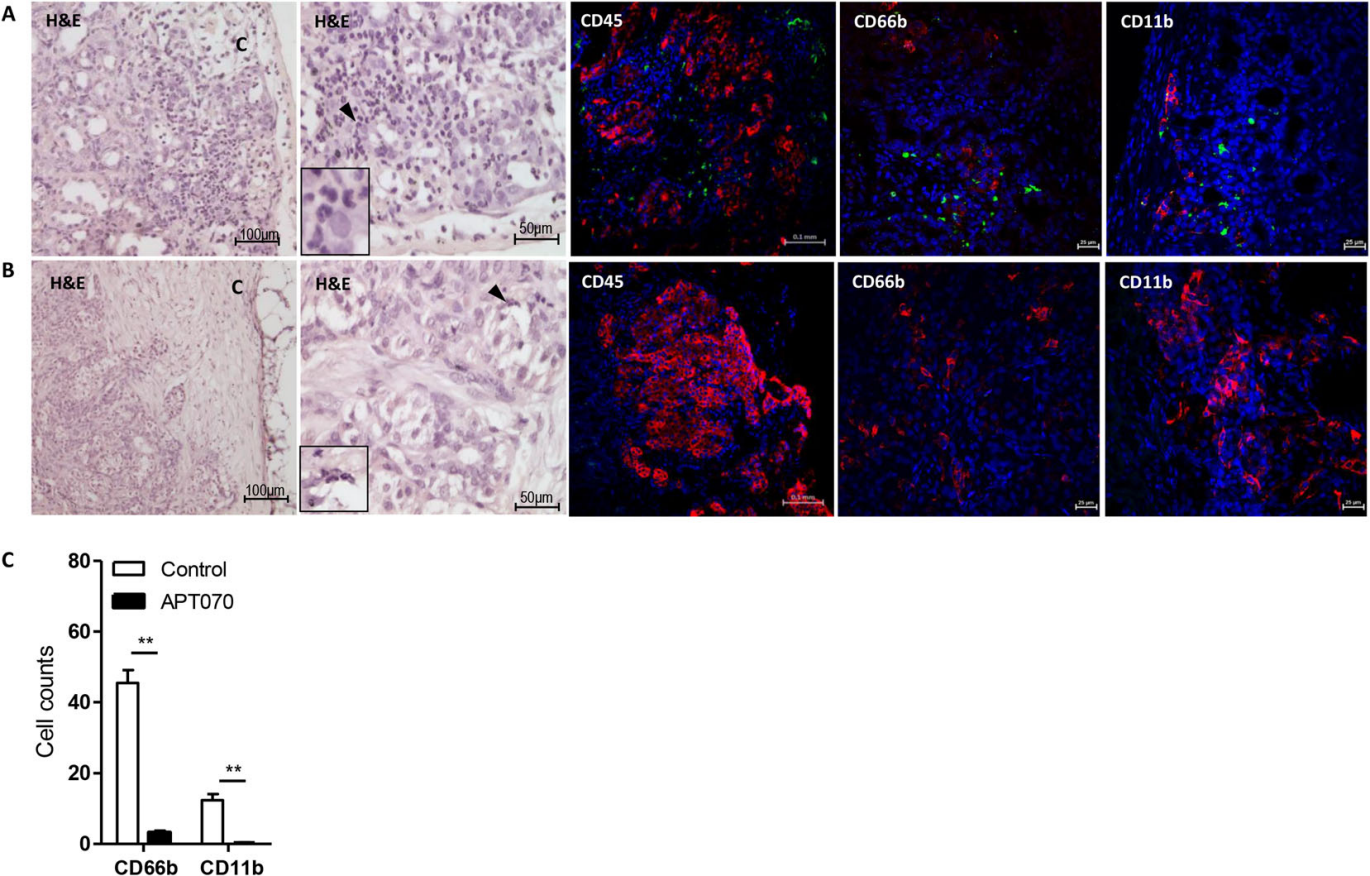
Infiltration of human leukocytes into human islet allografts from humanized NSG mice. Human islet grafts were harvested 5 days after transplantation and were fixed, embedded and sectioned. Samples were stained with H&E or with antibodies specific for human antigen: insulin (red), CD45 (green), CD66b (green), and CD11b (green). Nuclei were stained with DAPI (blue). HE staining shows massive infiltration of leukocytes in control grafts (A, H&E) compared to APT070 treated grafts (B, H&E). Images are representatives for control group (A) and APT070‐treated group (B). (C) Quantification of infiltration of CD66b and CD11b positive cells. *n* = 5 slides for each group. Data were represented as means ± SD of cell number in the field in amplifier power × 60. Cell numbers were counted in 3–5 fields in each slide and average was determined. H&E: haematoxylin and eosin staining. C: kidney capsule. Insets in the H&E staining images show enlarged area indicated by a black arrow.

Activated complement proteins C3a and C5a act as chemoattractants to recruit human leukocytes (Li and Zhou, 2013). To evaluate the inhibitory effect of APT070 on the production of these molecules, infiltration of human CD45^+^ cells, macrophages (CD11b^+^) and neutrophils (CD66b^+^) into graft‐bearing kidneys, 5 days after transplantation was examined. APT070‐treated islets displayed intact structure with decreased infiltration of these inflammatory cells, compared with controls (Figure 6).

### Pre‐incubation of islets with APT070 suppresses cytokine production in vitro and at early stages *in vivo*

**Figure 7:**
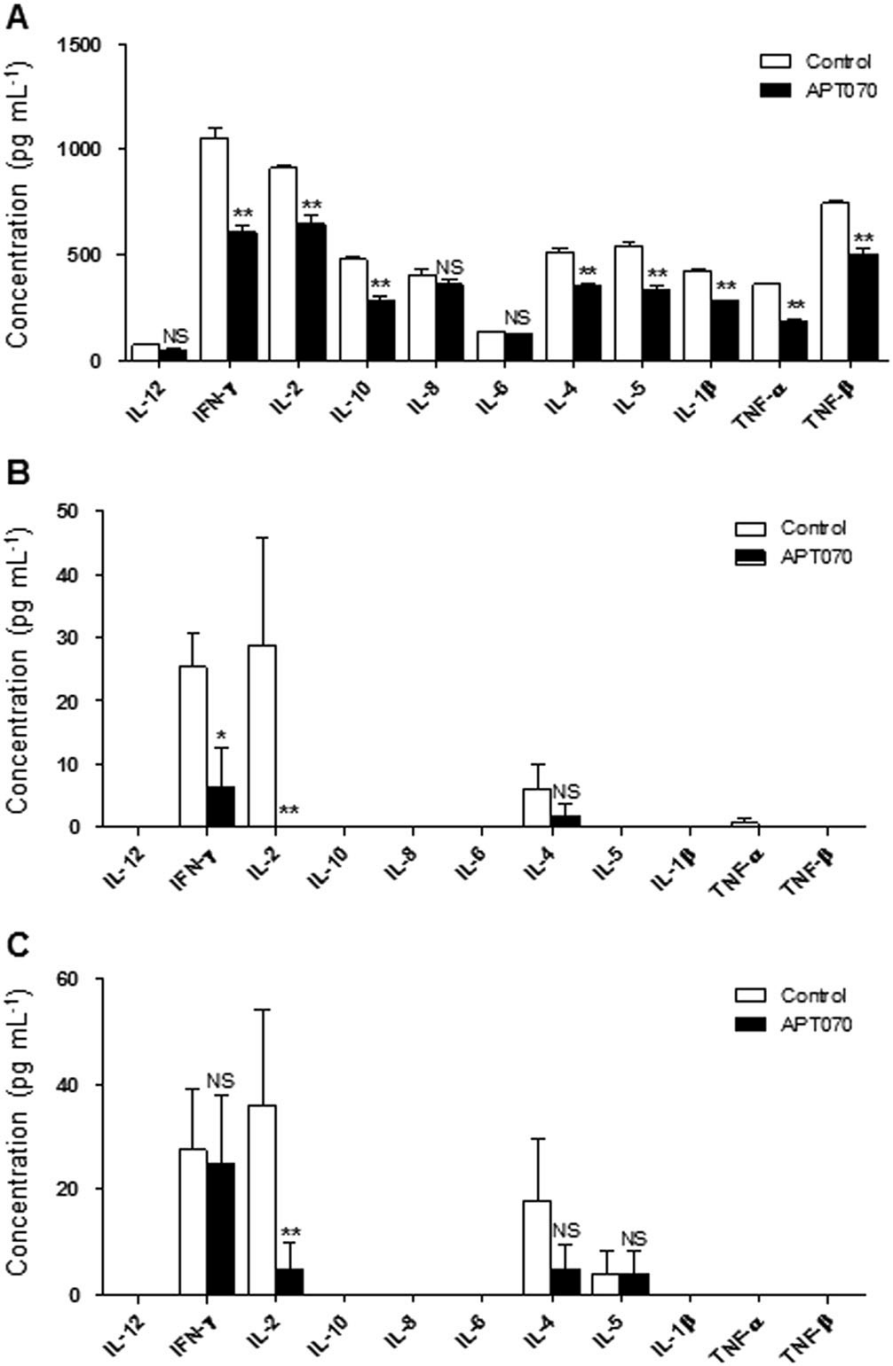
Supernatant and serum levels of cytokines. Supernatants were collected 30 min after exposure of islet to allogeneic whole blood (A). Sera were obtained from islet‐transplanted humanized NSG mice 5 (B) and 9 (C) days after transplantation. For supernatants, *n* = 6; for sera, *n* = 5 animals for each group. Values are mean ± SD. *P < 0.05. ** P < 0.01; significantly different as indicated. NS: no significant difference. Control islets had no treatment.

To further demonstrate the protective effect of APT070 on islet graft, human cytokine profiles were evaluated both *in vitro* and *in vivo*. A bead based Analyte Detection System was used for quantitative detection of human IFN‐γ, IL‐1β, IL‐2, IL‐4, IL‐5, IL‐6, IL‐8, IL‐10, TNF‐α and TNF‐β in the supernatants taken from the incubation of islets with blood, and in sera from islet‐transplanted humanized mice. Analysis revealed that treatment with APT070 significantly reduced levels of IFN‐γ, IL‐1β, IL‐2, IL‐4, IL‐5, IL‐10, TNF‐α and TNF‐β, with no effect on IL‐12, IL‐8 and IL‐6 (*n* = 6) (Figure 7A), following 30 minutes incubation.

*In vivo*, serum levels of human IFN‐γ and IL‐2 were significantly reduced in humanized mice 5 days after APT070 treated‐islet grafting, compared with untreated‐islet controls (*n* = 5 animals for each group) (Figure 7B), although IFN‐γ values were the same after 9 days. IL‐1β, IL‐6, IL‐8, IL‐10 and TNF‐α were undetectable (Figure 7C).

### Pre‐treatment of islets with APT070 induces earlier reversal of hyperglycaemia *in vivo*

**Figure 8:**
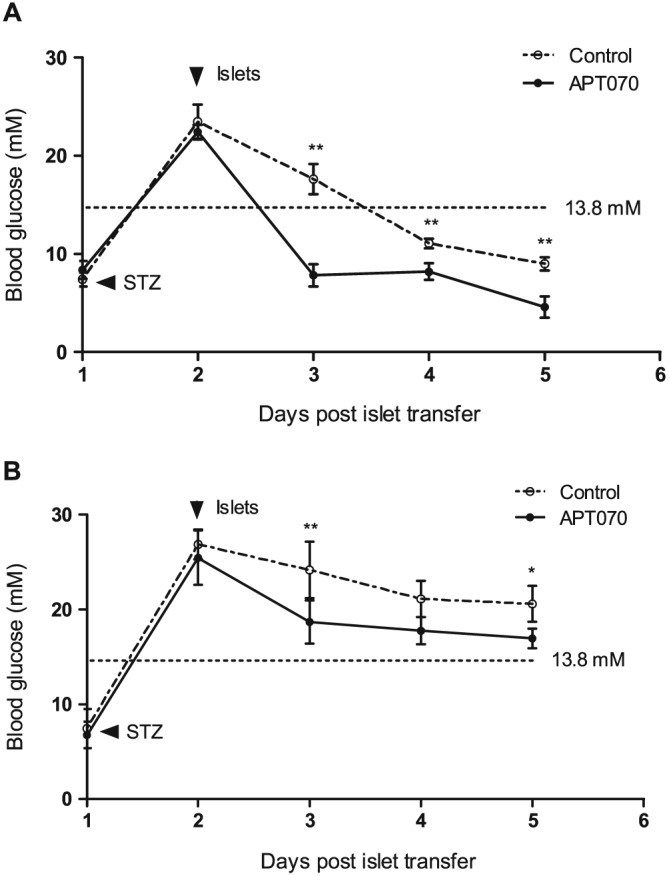
The plasma glucose level in the APT070‐treated and untreated human islet transferred into NSG (A) and hu‐NSG (B) mice rendered diabetic with streptozotocin. Tail blood was assessed for glucose after islet transplantation. *n* = 5 animals for each group. Data are expressed as means ± SD. *P < 0.05. **P < 0.01; significantly different as indicated. STZ: streptozotocin. Control islets had no treatment.

To correlate in vitro insulin secretion measurements with the ability of islets to restore normoglycemia *in vivo*, APT070‐treated islets were transplanted under the kidney capsule of diabetic hu‐NSG mice and NSG mice that did not receive CD34^+^ cells. Hyperglycaemia in both diabetic NSG (Figure 8A) and hu‐NSG mice (Figure 8B) was reversed earlier with APT070 treated islets, compared with the control islets, although normoglycaemia was not attained in hu‐NSG mice by the end of the experiment (5 days) (Xiao *et al.*, 2014). To determine the function of the transplant, the NSG mice underwent unilateral left nephrectomy at 60 days after transplantation. The data showed that a hyperglycemic state was restored within 24–48 hours after nephrectomy (Supplementary Figure 3).

## Discussion

In this study, it was found that a membrane‐localizing C3 convertase inhibitor (APT070) inhibited complement activation *in vitro* in a model mimicking IBMIR, and *in vivo* in humanized mice transplanted with human islets. The data demonstrated that APT070 protects human islet allografts from complement‐mediated damage through inhibition of infiltrates into grafts and regulation of cytokine production.

IBMIR is one of the main challenges faced by clinical islet transplantation and results in significant destruction of islets (Bennet *et al.*, 2000a; van der Windt *et al.*, 2007). IBMIR is believed to be a non‐specific inflammatory response related to the transplantation of islets directly to the blood microenvironment of the portal vein. It is probable that implanted islets express tissue factor (CD142) that activates the coagulation system. As a result, complement is activated and the islets are destroyed by innate factors. In the present study, the pathophysiological events triggered during direct exposure of human islets to an allogenic blood *in vitro*, mimicking clinical transplantation of human islets into the portal vein, have been characterised. Notably, the non‐physiological release of C‐peptide, which indicates massive beta cell death, was observed *in vitro* (Figure 5). This is consistent with the *in vivo* reports of auto‐, allo‐, and xeno‐islet transplantation (Moberg *et al.*, 2002; Mollnes *et al.*, 2002). Thus, incubation of human islets with allogenic blood has been proved to be a valuable model to investigate the events, as previously shown in a similar approach by van der Windt *et al.* (2012).

Incubation with anti‐coagulation agents leads to the surface modification of islets, which can inhibit IBMIR (Chen *et al.*, 2011; Teramura and Iwata, 2011). Different compounds targeting the complement system have been tested *in vitro* and/or *in vivo* to improve early islet survival in experimental models, such as the C5a inhibitory peptide (Tokodai *et al.*, 2010) and soluble complement receptor 1 (TP10) (Bennet *et al*., 2000b; Lundgren *et al.*, 2001). Although a high‐dose, systemic i.v. infusion of soluble complement receptor 1 (sCR1) (40 mg kg^−1^) has been shown to improve hyperacute xenograft rejection (Bennet, *et al.*, 2000b), the immobilization of sCR1 on islets was more effective than systemic infusion (Luan *et al.*, 2011). Like the immobilized sCR1, APT070 is a membrane‐bound protein, which inhibits complement activation at appropriate sites. When freshly isolated human islets were pretreated with APT070 (25 μg mL^−1^) for one hour, there was no a deleterious effect on islet secretory function (Figure 2). Glucose‐induced insulin secretion requires a depolarisation‐dependent influx of extracellular Ca^2^^+^ via voltage‐dependent calcium channels (VDCC) so the maintenance of normal secretory responses to glucose demonstrates that using the membrane bound APT070 did not adversely affect the beta cell membrane potential, nor the normal operation of VDCC. Similarly, the ability of pre‐treated islet to respond to the muscarinic cholineric agonist carbachol confirms that coating the islets with APT70 did not interfere with receptor expression or intracellular signalling pathways.

The present data also show that the pretreatment with APT070 resulted in a significant decrease in the release of C‐peptide and LDH from islets after incubation with human blood. Consistent with this, a peak serum level of C‐peptide can be associated with early islet cell death and/or islet destruction in patients with allo‐transplantation (Moberg *et al.*, 2002). The strategy of ‘painting’ APT070 on islets was also effective in preventing complement activation, as shown by the decreased production of soluble iC3b *in vitro* (Figure 3). Analysis of islet morphology revealed damaged islets embedded in blood clots with C4d and C5b‐9 deposited on the islet surface in untreated islets, but not in the APT070 pre‐treated group. This finding reflects clinical studies that suggest that C4d‐positive interacinar capillaries correlate with donor‐specific antibody‐mediated rejection in pancreas allografts (Torrealba *et al.*, 2008). Similar results have been obtained showing that APT070 completely prevented complement membrane attack complex formation at the nerve terminals in Miller Fisher Syndrome (Halstead *et al.*, 2005).

Expression of inflammatory mediators in isolated islets with immune modulating capacity could markedly influence the outcome of clinical islet transplantation (Johansson *et al.*, 2003). Use of APT070 has the potential to attenuate hyperinflammatory responses through reduction of local damage caused by the membrane attack complex (MAC) as well as by reduction of neutrophil activation directly through inhibition of C3a/C5a release and indirectly through inhibition of the complement‐mediated release of cytokines (Souza *et al.*, 2005). Our data showed that inhibition of the complement cascade by APT070 resulted in significantly decreased concentration of a number of pro‐inflammatory cytokines, including IFN‐γ, IL‐2, IL‐10, IL‐4, IL‐5, IL‐1β, TNF‐α and TNF‐β (Figure 7), consistent with a protective effect of APT070 against cytokine‐mediated damage to islet cells in the immediate post‐transplantation period. IL‐10 is a major protective endogenous cytokine in an islet xenograft rejection model (Yi *et al.*, 2012) and during intestinal reperfusion injury in rats (Souza *et al.*, 2003). Thus, it is clear that APT070 inhibits complement activation and islet damage by a mechanism other than by IL‐10 production. Interestingly, complement inhibition did not have an effect on IL‐8, which is the most relevant pro‐inflammatory cytokine found both in transplanted patients and in the murine counterpart after experimental islet transplant (Citro *et al.*, 2013). An explanation for this effect may lie in the time of sampling. Polymorphonuclear leukocytes (PMNs) and monocytes are the dominant cell types infiltrating the islet and producing cytokines *in vitro*, appearing in large numbers, 2 hours after incubation with ABO‐compatible blood (Moberg *et al.*, 2005), which is much later than our sampling time at 30 min. Equally beneficially, as IL‐6 protects pancreatic islet beta cells from pro‐inflammatory cytokine‐induced cell death and functional impairment *in vitro* and *in vivo* (Choi *et al.*, 2004), it is remarkable that APT070 had no effect on the production of IL‐6.

Immunodeficient mice that are engrafted with human functional cells (termed as humanized mice) are ideal preclinical models to investigate human immune responses in an *in vivo* setting (Brehm, *et al*., 2010a; Shultz *et al.*, 2007; King *et al.*, 2008). Non‐obese diabetic/severe combined immunodeficient mice harbouring a complete null mutation of IL‐2 receptor γ chain, NOD/SCID IL2r γ^null^ (NSG), are characterised by impairment in murine T‐, B‐ and NK‐cell development and function(Cao *et al.*, 1995; DiSanto *et al.*, 1995; Ohbo *et al.*, 1996), and can efficiently support the development of a functional human hemato‐lymphopoiesis (Brehm, *et al.*, 2010b; Ishikawa *et al.*, 2005; Watanabe *et al.*, 2009). Using the diabetic humanized NSG mouse model (Xiao *et al.*, 2014), we found that inhibition of the complement cascade by APT070 significantly decreased concentration of at least two pro‐inflammatory cytokines, IL‐2 and IFN‐γ (Figure 7). Whether APT070 had the same effects on cytokine profile *in vivo* are not known, because some human cytokines are undetectable in the humanized mice system (Xiao *et al.*, 2014). Nevertheless, in support of this concept, there was, at an early stage in the APT070‐treated animals, a marked inhibition of other cytokines, including TNF‐α, TNF‐b and IL‐6 (Souza *et al.*, 2005). The mechanisms underlying the inhibitory effects of APT070 on cytokines need to be further investigated. There are a number of limitations that prevent full use of our model system. In NSG mice, the human T cells are restricted to mouse major histocompatibility complex (MHC) and fail to interact productively with human antigen presenting cell within the host, leading to lack of class switching and immunoglobulin G (IgG) antibody production (Watanabe *et al.*, 2009; Xiao *et al.*, 2014). The generation of memory T cells in the model can also be problematic (Brehm *et al.*, 2013). This insufficient development of antigen‐specific immunity might explain why some of human cytokines could not be identified in our model system. To overcome these limitations, investigators have developed HLA‐transgenic NSG mice (Shultz *et al.*, 2010; Covassin *et al.*, 2011; Serra‐Hassoun *et al.*, 2014).

Using the hu‐NSG mice, we have recently shown a close relationship between complement C3d deposition and the damage of human islet allografts (Xiao *et al.*, 2014). The complement detected in this model system was human. The results suggested that complement might be involved in the destruction of islets even in the context of being placed under the kidney capsule, a site at which classical IBMIR does not occur (Xiao *et al.*, 2014). Now, using the same mouse model (Supplementary Figure S2), we have found that ATP070 markedly decreased complement deposition and infiltration of inflammatory cells at the early stage (Figure 4, 6), consistent with the *in vitro* data presented here. Consistently, APT070 treatment decreased serum levels of iC3b. These data indicate an inhibitory effect of APT070 on local production of complement. Indeed, within the graft, local production of complement contributes to non‐specific inflammation and parenchymal destruction (Brown *et al.*, 2006). It has been known for several years that innate immune cells, including macrophages and dendritic cells, can express and secrete a variety of complement components (Sacks *et al.*, 2009). These appear to be sufficient for the cleavage of C3 in the local inflammatory environment (Zhou *et al.*, 2006; Peng *et al.*, 2008). The data also suggested that pre‐incubation of human islet with APT070 may have partly affected reversal of hyperglycaemia *in vivo* in humanized NSG mice (Figure 8). Complete restoration of normoglycemia in APT070‐treated group was not observed, within the experimental period (5 days), whereas other studies (King *et al.*, 2008; Xiao *et al.*, 2014) showed that normoglycemia (blood glucose <13.8 mmol/l) was achieved after 5 days. Furthermore, the return of hyperglycemic state following removal of graft suggested the functionality of APT070‐treated islet *in vivo* (Supplementary Figure S3).

Given that the majority of the membrane‐localizing complement regulator was internalized by 40 h after intragraft delivery of APT070 (Patel *et al.*, 2006), the stability of APT070 in islets is a major obstacle that remains to be overcome. This may explain why the inhibitory action of APT070 on IFN‐γ production was lost after 9 days of transplantation (Figure 7). Ideally, APT070 should persist and control complement activation indefinitely (Patel *et al.*, 2006). Consequently, the possibility that combination therapy with APT070 can contribute to the permanent survival of human islet grafts is being explored.

In conclusion, this study demonstrates that APT070 prevented early islet damage involved in membrane leakage, activation of complement and deposition of the membrane attack complex *in vitro* and *in vivo*. Although the benefit did not translate into a statistically significant improvement of human islet allograft survival, the fact that both cytokine levels and tissue damage were considerably improved indicates the importance of APT070 as a potential therapeutic tool. These data might provide a rationale for considering clinical trials of APT070 in human islet transplantation, and other complement dependent disorders.

## Conflict of interest

The authors declare no conflicts‐of‐interest.

## Authorship contribution statement

F. X. and L. M. performed the research; M. Z. and A. P. helped in *in vitro* viability and functional assessment of the islets; F. X., L. M., R. L. and G. L. designed the research study; R. A. S., G. C. H., P. M. J. and S. P. contributed essential reagents and tools; F. X. and L. M. analysed the data; L. M., A. D., R. A. S., P. M. J, R. L. and G. L. edited the manuscript; F. X. wrote the paper.

## Supporting information


**Figure 1** Assessment of APT070‐treated islet viability. Human islets were treated with APT070 of serial dilutions of 0.4 µM to 0.16 µM, and then stained with fluorescein diacetate (green) and propidium iodide (red). Control islets received no treatment. Inset images show enlarged area indicated by a white arrow. Green and red colours indicate live and dead cells respectively.
**Figure 2** Flow cytometry analysis of human cell grafts three months after CD34^+^ stem cell reconstitution in NSG mice. Each plot represents one mouse. (A) The percentage of human cell engraftment in the peripheral blood 16 weeks after CD34^+^ stem cell injection. (B) Representative plot of flow cytometry analysis.
**Figure 3** Non‐fasted plasma glucose level over time in islet transplanted NSG mice rendered diabetic with streptozotocin. Values are shown as means ± SD. N = 3 animals for each group. Nephrectomy: graft‐bearing kidney was removed. Normoglycemia was defined as ≤ 13.8 mM glucose in plasma.

Supporting info item

Supporting info item

Supporting info item

Supporting info item
