# Supplementary figures and images for: APT070 (mirococept), a membrane‐localizing C3 convertase inhibitor, attenuates early human islet allograft damage in vitro and in vivo in a humanized mouse model
Source: Br J Pharmacol. 2016 Jan 11;173(3):575–87. doi: 10.1111/bph.13388 (PMC4728428; doi:10.1111/bph.13388)

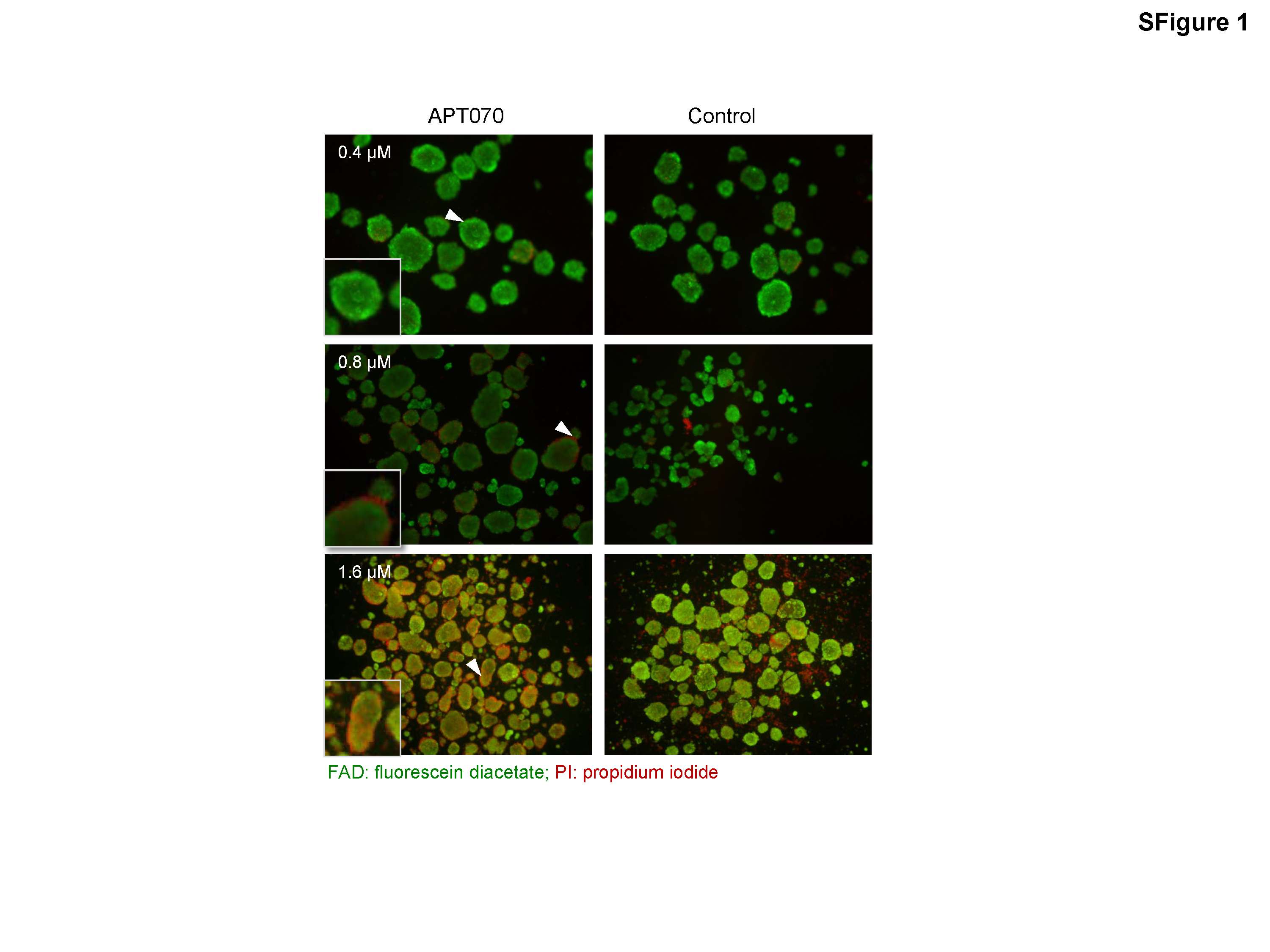

Supplement: Supplementary file 1 — Supporting info item [file BPH-173-575-s001.tif]

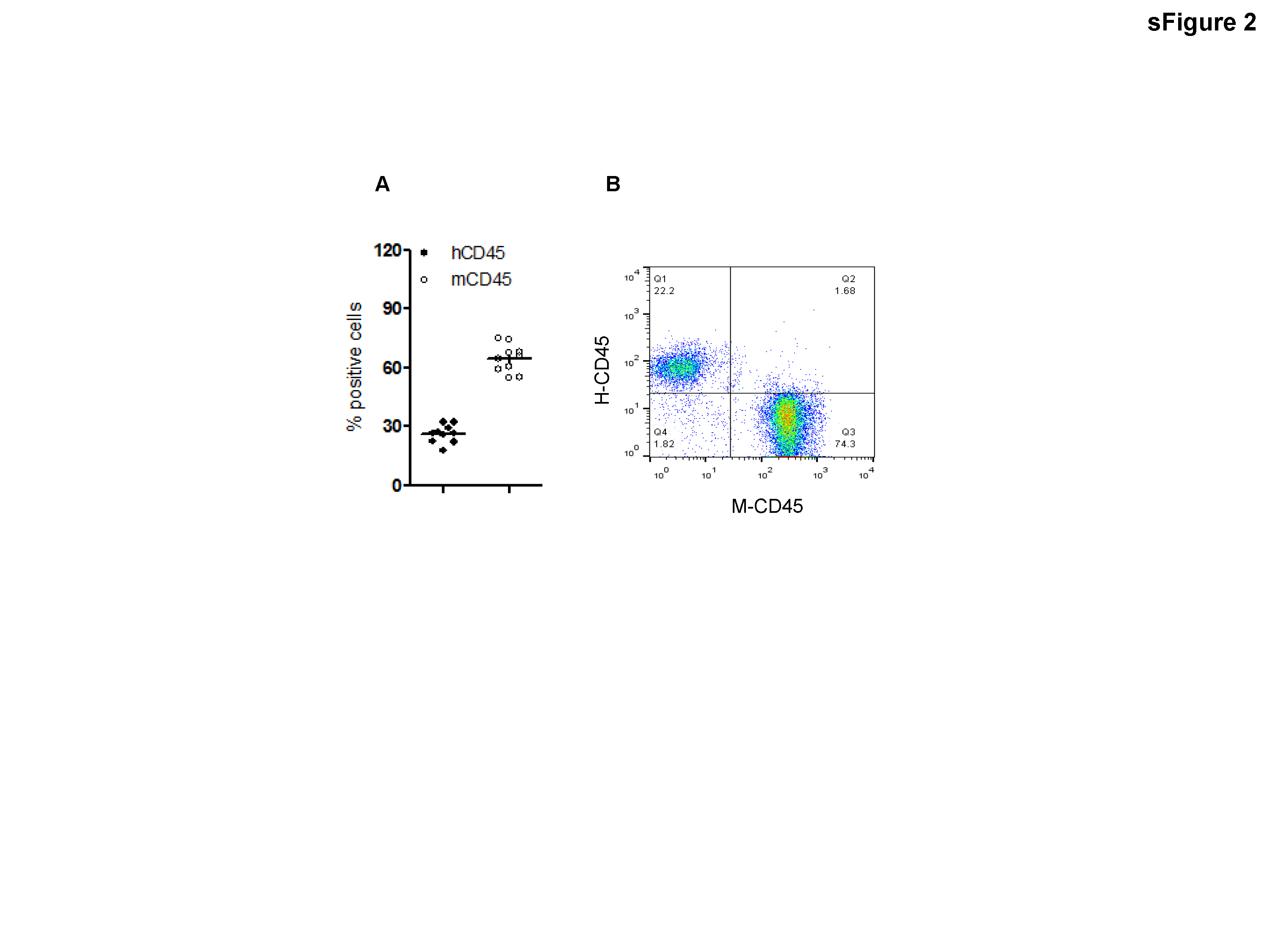

Supplement: Supplementary file 2 — Supporting info item [file BPH-173-575-s002.tif]

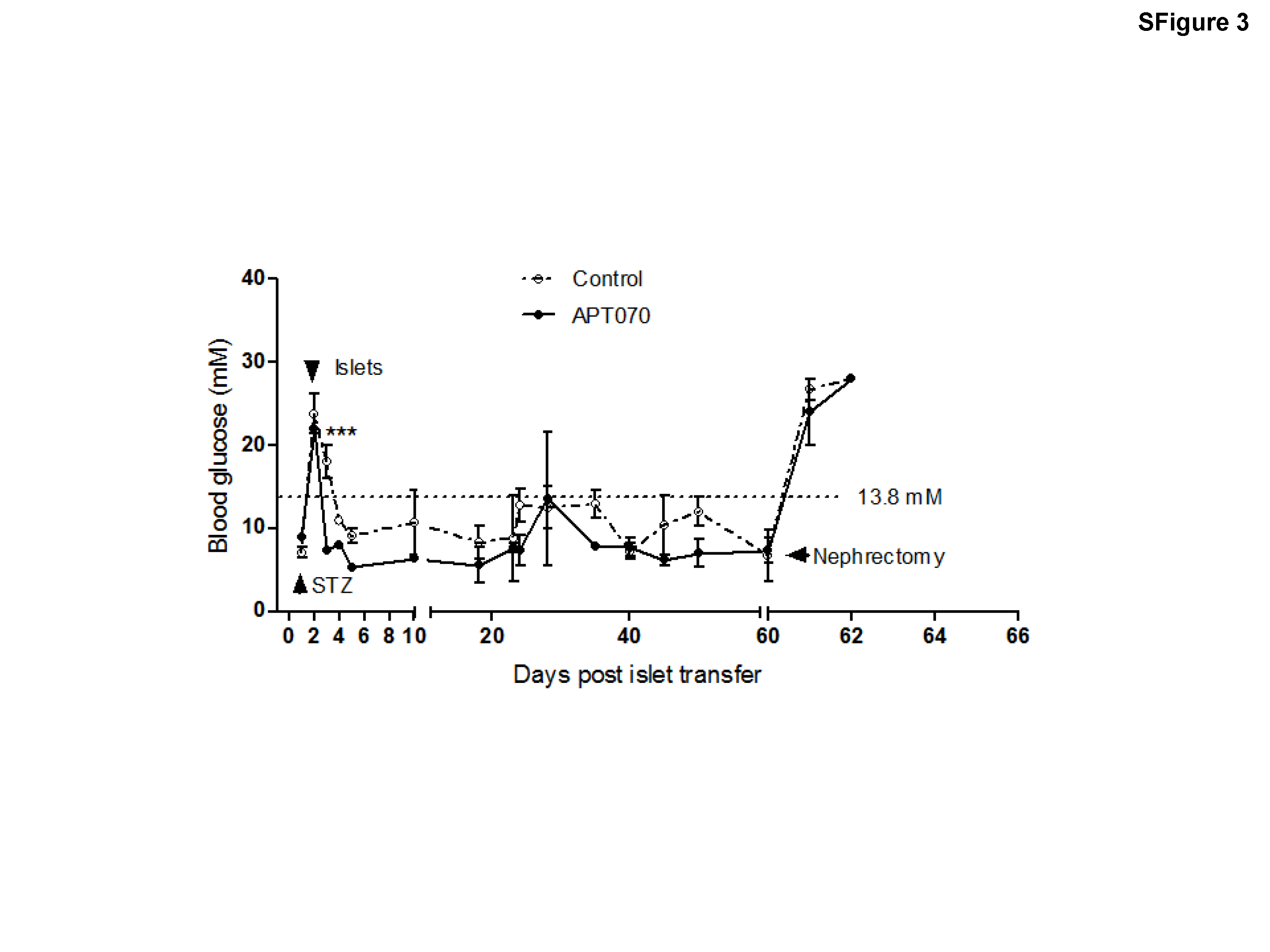

Supplement: Supplementary file 3 — Supporting info item [file BPH-173-575-s003.tif]
