# Supplementary material for: APT070 (mirococept), a membrane‐localizing C3 convertase inhibitor, attenuates early human islet allograft damage in vitro and in vivo in a humanized mouse model
Source: Br J Pharmacol. 2016 Jan 11;173(3):575–87. doi: 10.1111/bph.13388 (PMC4728428; doi:10.1111/bph.13388)
Supplement: Supplementary file 4 — Supporting info item [file BPH-173-575-s004.docx]

**Supplementary Figure legends**

**sFigure 1:** Assessment of APT070-treated islet viability. Human islets were treated with APT070 of serial dilutions of 0.4 μM to 0.16 uM, and then stained with fluorescein diacetate (green) and propidium iodide (red). Control islets received no treatment. Inset images show enlarged area indicated by a white arrow. Green and red colours indicate live and dead cells respectively.

**sFigure 2:** Flow cytometry analysis of human cell grafts three months after CD34 stem cell reconstitution in NSG mice. Each plot represents one mouse. (A) The percentage of human cell engraftment in the peripheral blood 16 weeks after CD34^+^ stem cell injection. (B) Representative plot of flow cytometry analysis.

**sFigure 3**: Non-fasted plasma glucose level over time in islet transplanted NSG mice rendered diabetic with streptozotocin. Values are shown as means ± SD. N = 3 animals for each group. Nephrectomy: graft-bearing kidney was removed. Normoglycemia was defined as 13.8 mM glucose in plasma.
